# Supplementary material for: Relationship of Training Factors and Resilience with Injuries in Ski Mountaineers
Source: Sports (Basel). 2022 Nov 25;10(12):191. doi: 10.3390/sports10120191 (PMC9782121; doi:10.3390/sports10120191)
Supplement: Supplementary file 1 [file sports-10-00191-s001.zip › sports-1982974-supplementary.pdf]

Ski Mountaineering

This questionnaire was created in order to discover and investigate in depth the type of injuries suffered by skiers, as well as the most influential variables in these injuries.

The processing, communication and transfer of the personal data of all participants will comply with the provisions of Organic Law 3/2018, of 5 December, on the Protection of Personal Data and the guarantee of digital rights. In accordance with the provisions of the aforementioned legislation, you may exercise your right to oppose and cancel your data by contacting the directors of the study. The researchers of the project may have access to the volunteer's data. The personal data and information obtained from this study, with guaranteed privacy for your identity, will be known only by the researchers of the project. By accepting this consent you authorise the collection, storage and analysis of your requested data, unlinked to your identity by a double reversible coding system. Those who continue the process will show their acceptance to the characteristics of the research and will ensure their understanding.

* Required

1. 0.- I have read and I accept the terms and conditions. *

   Mark only one oval.

   ◯ Yes

   ◯ No

2. 1.- Country of residence *

   ____

3. 2.- Date of birth *

   ____

   Example: January 7, 2019

4. 3.- Gender *

Mark only one oval.

- Female
- Male
- Other

5. 4.- Weight in kg *

____

6. 5.- Height in cm *

____

7. 6.- Do you smoke? *

Mark only one oval.

- Yes
- No
- Sporadically

8. 7.- Do you drink alcoholic beverages? *

Mark only one oval.

- Yes
- No
- Sporadically

9. 7.1.- If the answer above was yes, approximately how many "doses" per week?

Mark only one oval.

- 1
- 2
- 3
- 4
- 5
- 6
- 7
- 8
- 9
- 10
- More than 10

10. 8.- How many years have you been ski mountaineering? *

11. 9.- In case you are federated, how many years have you been federated? (If you are not federated, indicate "NO").

12. 10.- Do you compite in ski mountaineering? *

Mark only one oval.

- Yes
- No (continue to question 13)

13. 11.- If you answered yes: in which modalities of ski mountaineering do you compete?

Check all that apply.

- [ ] Sprint
- [ ] Vertical
- [ ] Individual
- [ ] Relays
- [ ] Team
- [ ] Others

14. 11.1.-If you have chosen other, please indicate which one. 0 points

____

15. 12.-How many competitions do you do per year?

Mark only one oval.

- I do not compete.
- 1-5
- 6-10
- 11-15
- 16-20
- 21-25
- 26-30
- 31-35
- 36-40
- More than 40

16. 12.1.- How many of these competitions are international?

____

17. 13.- How many days per week do you usually ski? *

Mark only one oval.

1
2
3
4
5
6
7

18. 14.- How many training sessions do you usually do per day? *

Mark only one oval.

1
2
3

19. 15.- On average, how many hours do you train each ski training session? *

Mark only one oval.

- 1h
- 1,5h
- 2h
- 2,5h
- 3h
- 3,5h
- 4h
- 4,5h
- 5h
- 5,5h
- 6h

20. 16.- What is the volume (time) of the sessions that you have skied the longest? *

Mark only one oval.

- 1h
- 2h
- 3h
- 4h
- 5h
- 6h
- More than 6h

21. 17.- Do you complement your ski mountaineering training with any other sport? *

Mark only one oval.

- Yes
- No

22. 17.1.- If the answer to the previous question was yes: which one?

23. 17.2.-How many times per week?

Mark only one oval.

1	⬭
2	⬭
3	⬭
4	⬭
5	⬭
6	⬭
7	⬭

24. 18.- Do you complement your ski mountaineering sessions with strength training in the gym? *

Mark only one oval.

⬭ Yes

⬭ No (go to question 18)

25. 18.1.- If the answer to the previous question was yes: how many times per week?

Mark only one oval.

1	⬭
2	⬭
3	⬭
4	⬭
5	⬭
6	⬭
7	⬭

26. 18.2.- How many months per year do you do strength training? *

Mark only one oval.

- 1
- 2
- 3
- 4
- 5
- 6
- 7
- 8
- 9
- 10
- 11
- 12

27. 18.3.- What kind of strength training do you usually do in the gym in the first part of the season or pre-season?

Check all that apply.

- [ ] Less than 6 repetitions with a weight that allows me to reach only that number of repetitions
- [ ] Between 6 and 10 repetitions with a weight that allows me to reach only that number of repetitions
- [ ] More than 10 repetitions with a weight that allows me to reach only that number of repetitions
- [ ] More than 15 repetitions with a weight that allows me to reach more repetitions (not only that number of repetitions)
- [ ] Less than 10 repetitions with priority given to performing them as explosively or quickly as possible

28. 18.4.- What kind of strength training do you usually do in the gym during the season?

Mark only one oval.

- Less than 6 repetitions with a weight that allows me to reach only that number of repetitions
- Between 6 and 10 repetitions with a weight that allows me to reach only that number of repetitions
- More than 10 repetitions with a weight that allows me to reach only that number of repetitions
- More than 15 repetitions with a weight that allows me to reach more repetitions (not only that number of repetitions)
- Less than 10 repetitions with priority given to performing them as explosively or quickly as possible

29. 19.- Do you complement your ski mountaineering sessions with core work? *

Mark only one oval.

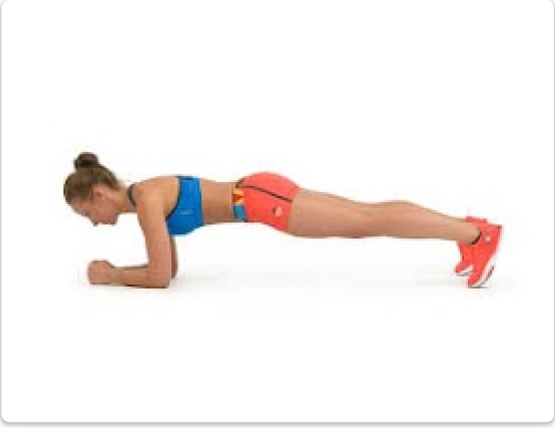

- Yes
- No

30. 19.1.- If the answer to the previous question was yes: how many sessions per week?

Mark only one oval.

1 ⬭
2 ⬭
3 ⬭
4 ⬭
5 ⬭
6 ⬭
7 ⬭
8 ⬭
9 ⬭
10 ⬭

31. 20.- Do you complement your ski mountaineering sessions with stretching and flexibility exercises? *

Mark only one oval.

- Yes
- No
- Sometimes

32. 20.1.- If you answered yes to the previous question: how many times per week?

Mark only one oval.

- 1
- 2
- 3
- 4
- 5
- 6
- 7

33. 20.2.- What kind of stretching do you do?

Mark only one oval.

- I hold a position for a few seconds.
- Joint mobility
- Other

34. 20.3.- If you answered other, please indicate which one:

____

35. 20.4.- At what moment or moments during the session do you stretch?

Check all that apply.

- [ ] Before
- [ ] During the session
- [ ] After

36. 21.- Do you take care of your diet? *

Mark only one oval.

- Yes
- No
- Sometimes

37. 22.- You have at your disposal: *

Check all that apply.

- [ ] Physiotherapist
- [ ] Physical trainer
- [ ] Sports doctor
- [ ] Nutritionist
- [ ] None (I train myself)
- [ ] Others

38. 22.1.- If you have chosen others indicate which one:

39. 23.- How many pairs of skis do you own? *

Mark only one oval.

- 1
- 2
- 3
- 4
- 5
- More than 5

40. 23.1.- How wide are the skis you usually use? *

41. 24.- How many pairs of ski poles do you have? *

Mark only one oval.

- 1
- 2
- 3
- 4
- 5
- More than 5

42. 24.1.- What length of ski poles do you usually use? *

43. 24.2.- What material are the walking sticks you usually use? *

Mark only one oval.

- Carbon
- Aluminium
- Titanium
- Other

44. 24.3.- If you answered other in the previous question, please indicate which one:

____

45. 25.-What material are the boots you usually wear? *

Mark only one oval.

- Carbon
- Plastic
- Mixed, plastic lower part, carbon cuff
- Other

46. 25.1.- If you answered other in the previous question, please indicate which one:

____

26.- Items

From 0- Never to 4- Very often

47. 26.1.- During the last month... [How often have you felt unable to control the important things in your life?] *

Mark only one oval.

- 0- never
- 1- rarely
- 2- from time to time
- 3-often
- 4- very often

48. 26.2.- In the last month... [How often have you been confident about your ability to handle your personal problems?] *

Mark only one oval.

- 0- never
- 1- rarely
- 2- from time to time
- 3- often
- 4- very often

49. 26.3.- In the last month... [How often have you felt that things are going well for you?] *

Mark only one oval.

- 0- never
- 1- rarely
- 2- from time to time
- 3- often
- 4- very often

50. 26.4.- In the last month... [How often have you felt that difficulties accumulate so much that you can't overcome them?] *

Mark only one oval.

- 0- never
- 1- rarely
- 2- from time to time
- 3- often
- 4- very often

27.- Three items

From 0 to 10

51. 27.1. My thoughts are: *

Mark only one oval.

RELAXED
0
1
2
3
4
5
6
7
8
9
10
WORRIED

52. 27.2. My body feels: *

Mark only one oval.

	RELAXED
0	⬭
1	⬭
2	⬭
3	⬭
4	⬭
5	⬭
6	⬭
7	⬭
8	⬭
9	⬭
10	⬭
	TENSE

53. 27.3. I feel: *

Mark only one oval.

	CONFIDENT
0	⬭
1	⬭
2	⬭
3	⬭
4	⬭
5	⬭
6	⬭
7	⬭
8	⬭
9	⬭
10	⬭
	AFRAID

All questions will have an answer option from 1 to 5, where 1 is never and 5 is almost always.

54. 28.- I am able to adapt when changes occur. *

Mark only one oval.

1
2
3
4
5

55. 29.- I can cope with anything. *

Mark only one oval.

1
2
3
4
5

56. 30.- I try to see the funny side of things when faced with problems. *

Mark only one oval.

1
2
3
4
5

57. 31.- Facing difficulties can make me stronger. *

Mark only one oval.

1
2
3
4
5

58. 32.- I have a tendency to recover quickly after illness, injury or other deprivation. *

Mark only one oval.

1
2
3
4
5

59. 33.- I believe that I can achieve my goals, even if there are obstacles. *

Mark only one oval.

1
2
3
4
5

60. 34.- Under pressure, I focus and think clearly. *

Mark only one oval.

1
2
3
4
5

61. 35.- I am not easily discouraged by failure. *

Mark only one oval.

1
2
3
4
5

62. 36.- I believe that I am a strong person when faced with challenges and difficulties of life. *

Mark only one oval.

1 ◯
2 ◯
3 ◯
4 ◯
5 ◯

63. 37.- I am able to handle unpleasant and painful feelings such as sadness, fear and anger. *

Mark only one oval.

1 ◯
2 ◯
3 ◯
4 ◯
5 ◯

64. 38.- Have you suffered any other discomfort or injury while ski mountaineering? *

Mark only one oval.

- Yes
- No *Skip to question 121*

1

65. 39.1.- When did this injury occur? *

Mark only one oval.

- In the last 6 months
- In the last 12 months
- In the last 18 months
- In the last 24 months
- More than 24 months

66. 39.1.1.- If the injury occurred more than 24 months ago, please indicate when:

67. 39.2.- Anatomical region in which the injury occurred: *

Mark only one oval.

- Hands
- Wrists
- Forearm
- Elbow
- Arm
- Shoulder
- Clavicle
- Neck
- Spine- Lower back
- Spine- Upper back
- Ribs
- Chest
- Abdomen
- Hip
- Pelvis
- Fingers
- Thigh
- Knee
- Leg
- Ankle
- Foot
- Nails
- Others
- Other: ______________________

68. 39.3.- On which side of the body? *

Mark only one oval.

- Right side
- Left side
- Centre

69. 39.4.- At what point in time did it occur? *

Mark only one oval.

- Warm up
- Training
- Cool down
- Competition

70. 39.5.- At what point in the activity did it take place? *

Mark only one oval.

- Uphill
- Downhill
- Other

71. 39.5.1.- If in the previous question the answer was other, please indicate which one:

____

72. 39.6.- Why did it happen? *

Mark only one oval.

- Impact (due to a blow)
- Overuse (related to the type and volume of training load)

73. 39.7.- What type of injury was it? *

Mark only one oval.

- New injury
- Relapse

74. 39.8.- What was the diagnosis? *

Mark only one oval.

- Contusion
- Dislocation
- Sprain
- Fracture
- Superficial wound
- Irritation
- Burn
- Muscle rupture
- Muscle contracture
- Tendinitis
- Fissure
- Muscular micro rupture
- Others
- Other: ______________________

75. 39.9.- Who treated your injury? *

Check all that apply.

- [ ] Doctor
- [ ] Physiotherapist
- [ ] Coach
- [ ] Massage therapist
- [ ] Osteopath
- [ ] Other

76. 39.9.1.- If you have chosen another one indicate which one:

____

77. 39.10.- How long were you out of skiing because of the injury? *

Mark only one oval.

- 1-3 days
- 4-7 days
- 1 to 2 weeks
- 2 weeks to 1 month
- 1 to 3 months
- More than 3 months
- Other: _______________

78. 39.11.- Have you suffered any other discomfort or injury while ski mountaineering? *

Mark only one oval.

- Yes
- No *Skip to question 121*

2

79. 39.1.- When did this injury occur? *

Mark only one oval.

- In the last 6 months
- In the last 12 months
- In the last 18 months
- In the last 24 months
- More than 24 months

80. 39.1.1.- If the injury occurred more than 24 months ago, please indicate when:

____

81. 39.2.- Anatomical region in which the injury occurred: *

Mark only one oval.

- Hands
- Wrists
- Forearm
- Elbow
- Arm
- Shoulder
- Clavicle
- Neck
- Spine- Lower back
- Spine- Upper back
- Ribs
- Chest
- Abdomen
- Hip
- Pelvis
- Fingers
- Thigh
- Knee
- Leg
- Ankle
- Foot
- Nails
- Others
- Other: ________________________________

82. 39.3.- On which side of the body? *

Mark only one oval.

- Right side
- Left side
- Centre

83. 39.4.- At what point in time did it occur? *

Mark only one oval.

- Warm up
- Training
- Cool down
- Competition

84. 39.5.- At what point in the activity did it take place? *

Mark only one oval.

- Uphill
- Downhill
- Other

85. 39.5.1.- If in the previous question the answer was other, please indicate which one:

____

86. 39.6.- Why did it happen? *

Mark only one oval.

- Impact (due to a blow)
- Overuse (related to the type and volume of training load)

87. 39.7.- What type of injury was it? *

Mark only one oval.

- New injury
- Relapse

88. 39.8.- What was the diagnosis? *

Mark only one oval.

- Contusion
- Dislocation
- Sprain
- Fracture
- Superficial wound
- Irritation
- Burn
- Muscle rupture
- Muscle contracture
- Tendinitis
- Fissure
- Muscular micro rupture
- Others
- Other: ____________

89. 39.9.- Who treated your injury? *

Check all that apply.

- Doctor
- Physiotherapist
- Coach
- Massage therapist
- Osteopath
- Other

90. 38.9.1.- If you have chosen another one indicate which one:

____

91. 39.10.- How long were you out of skiing because of the injury? *

Mark only one oval.

- 1-3 days
- 4-7 days
- 1 to 2 weeks
- 2 weeks to 1 month
- 1 to 3 months
- More than 3 months
- Other: _______________

92. 39.11.- Have you suffered any other discomfort or injury while ski mountaineering? *

Mark only one oval.

- Yes
- No *Skip to question 121*

3

93. 39.1.- When did this injury occur? *

Mark only one oval.

- In the last 6 months
- In the last 12 months
- In the last 18 months
- In the last 24 months
- More than 24 months

94. 39.1.1.- If the injury occurred more than 24 months ago, please indicate when:

____

95. 39.2.- Anatomical region in which the injury occurred: *

Mark only one oval.

- Hands
- Wrists
- Forearm
- Elbow
- Arm
- Shoulder
- Clavicle
- Neck
- Spine- Lower back
- Spine- Upper back
- Ribs
- Chest
- Abdomen
- Hip
- Pelvis
- Fingers
- Thigh
- Knee
- Leg
- Ankle
- Foot
- Nails
- Others
- Other: _______________

96. 39.3.- On which side of the body? *

Mark only one oval.

- Right side
- Left side
- Centre

97. 39.4.- At what point in time did it occur? *

Mark only one oval.

- Warm up
- Training
- Cool down
- Competition

98. 39.5.- At what point in the activity did it take place? *

Mark only one oval.

- Uphill
- Downhill
- Other

99. 39.5.1.- If in the previous question the answer was other, please indicate which one:

____

100. 39.6.- Why did it happen? *

Mark only one oval.

- Impact (due to a blow)
- Overuse (related to the type and volume of training load)

101. 39.7.- What type of injury was it? *

Mark only one oval.

- New injury
- Relapse

102. 39.8.- What was the diagnosis? *

Mark only one oval.

- Contusion
- Dislocation
- Sprain
- Fracture
- Superficial wound
- Irritation
- Burn
- Muscle rupture
- Muscle contracture
- Tendinitis
- Fissure
- Muscular micro rupture
- Others
- Other: ______________________

103. 39.9.- Who treated your injury? *

Check all that apply.

- [ ] Doctor
- [ ] Physiotherapist
- [ ] Coach
- [ ] Massage therapist
- [ ] Osteopath
- [ ] Other

104. 38.9.1.- If you have chosen another one indicate which one:

____

105. 39.10.- How long were you out of skiing because of the injury? *

Mark only one oval.

- 1-3 days
- 4-7 days
- 1 to 2 weeks
- 2 weeks to 1 month
- 1 to 3 months
- More than 3 months
- Other: ______________________________

106. 39.11.- Have you suffered any other discomfort or injury while ski mountaineering? *

Mark only one oval.

- Yes
- No *Skip to question 121*

4

107. 39.1.- When did this injury occur? *

Mark only one oval.

- In the last 6 months
- In the last 12 months
- In the last 18 months
- In the last 24 months
- More than 24 months

108. 39.1.1.- If the injury occurred more than 24 months ago, please indicate when:

____

109. 39.2.- Anatomical region in which the injury occurred: *

Mark only one oval.

- Hands
- Wrists
- Forearm
- Elbow
- Arm
- Shoulder
- Clavicle
- Neck
- Spine- Lower back
- Spine- Upper back
- Ribs
- Chest
- Abdomen
- Hip
- Pelvis
- Fingers
- Thigh
- Knee
- Leg
- Ankle
- Foot
- Nails
- Others
- Other: _______________________________

110. 39.3.- On which side of the body? *

Mark only one oval.

- Right side
- Left side
- Centre

111. 39.4.- At what point in time did it occur? *

Mark only one oval.

- Warm up
- Training
- Cool down
- Competition

112. 39.5.- At what point in the activity did it take place? *

Mark only one oval.

- Uphill
- Downhill
- Other

113. 39.5.1.- If in the previous question the answer was other, please indicate which one:

____

114. 39.6.- Why did it happen? *

Mark only one oval.

- Impact (due to a blow)
- Overuse (related to the type and volume of training load)

115. 39.7.- What type of injury was it? *

Mark only one oval.

- New injury
- Relapse

116. 39.8.- What was the diagnosis? *

Mark only one oval.

- Contusion
- Dislocation
- Sprain
- Fracture
- Superficial wound
- Irritation
- Burn
- Muscle rupture
- Muscle contracture
- Tendinitis
- Fissure
- Muscular micro rupture
- Others
- Other: __________

117. 39.9.- Who treated your injury? *

Check all that apply.

- Doctor
- Physiotherapist
- Coach
- Massage therapist
- Osteopath
- Other

118. 38.9.1.- If you have chosen another one indicate which one:

____

119. 39.10.- How long were you out of skiing because of the injury? *

Mark only one oval.

- 1-3 days
- 4-7 days
- 1 to 2 weeks
- 2 weeks to 1 month
- 1 to 3 months
- More than 3 months
- Other: _______________________________

120. 39.11.- Have you suffered any other discomfort or injury while ski mountaineering? *

Mark only one oval.

- Yes
- No *Skip to question 121*

121. 43.- Do you suffer from any chronic pain or discomfort related to the sport of ski mountaineering? *

Mark only one oval.

- Yes
- No (go to question 44)

122. 43.1. If you have answered yes, please indicate where:

____

123. 43.2.- If you answered yes, when does the pain occur?

Mark only one oval.

- While ski mountaineering
- When you are not ski mountaineering
- In both of the above situations

124. 43.3.- What do you think this is due to?

Mark only one oval.

- Poor preparation
- It is an old injury
- Training loads (too much training)
- Other reasons
- Other: ______________________

125. 43.3.1.- If you have chosen other reasons, please indicate which ones:

____

126. 44.- Do you always warm up in your training sessions?

Mark only one oval.

- Yes
- No
- Sometimes

127. 45.- Do you always do a cool down in your training sessions?

Mark only one oval.

- Yes
- No
- Sometimes

128. 46.- Do you follow any injury prevention protocol? *

Mark only one oval.

- Yes
- No

129. 46.1.- If you answered yes, please indicate how many days per week:

Mark only one oval.

- 1
- 2
- 3
- 4
- 5
- 6
- 7

Thank you very much for your participation and contribution to this study.

130. If you are interested in receiving the results of this research please specify your EMAIL below:

131. THANK YOU VERY MUCH FOR YOUR TIME. If you have any comments or clarifications you would like to make about the questionnaire, please use the following space (if you have any questions please do not hesitate to contact us: arkaitz.castaneda@deusto.es)

This content is neither created nor endorsed by Google.

Google Forms
